# Application of the ant colony optimization algorithm for the construction of a short version of the German alcohol decisional balance scale

**DOI:** 10.1038/s41598-025-12087-3

**Published:** 2025-07-25

**Authors:** Anne Moehring, Christian Meyer, Ulrich John, Hans-Juergen Rumpf, Gallus Bischof, Jennis Freyer-Adam, Sophie Baumann, Andreas Staudt

**Affiliations:** 1 Department of Methods in Community Medicine, Institute of Community Medicine, University Medicine Greifswald, Greifswald, Germany; 2 DZHK (German Center for Cardiovascular Research), Partner Site Greifswald, Greifswald, Germany; 3 Department of Prevention Research and Social Medicine, Institute of Community Medicine, University Medicine Greifswald, Greifswald, Germany; 4 Department of Psychiatry and Psychotherapy, Research Group S:TEP, University of Luebeck, Luebeck, Germany; 5 Institute and Policlinic of Occupational and Social Medicine, Technische University Dresden, Dresden, Germany

**Keywords:** Short scale construction, Ant colony optimization algorithm, Decisional balance, Reliability, Validity, Psychology, Population screening

## Abstract

Self-report questionnaires must be psychometrically sound, but also brief and efficient to avoid participant nonresponse and fatigue, especially in the health and prevention sciences. Meta-heuristics such as the Ant Colony Optimization (ACO) algorithm overcome limitations of the traditional stepwise approach of selecting items based on few or a single statistical criterion. The aim of this paper was to demonstrate the use of the ACO algorithm by constructing a short version of the German Alcohol Decisional Balance Scale (ADBS). Self-report data from three studies (*N* = 1,834; 19% women; mean age = 31.4 years) was used that proactively recruited alcohol consumers from the general population and general hospitals in Germany. All participants rated the perceived importance of different pros and cons in their decision to drink alcohol (decisional balance) on a 5-point Likert scale. Optimizing different model fit indices and theoretical considerations simultaneously, the ACO algorithm produced a psychometrically valid and reliable 10-item short scale that was superior to the 26-item full ADBS scale and an already established 10-item short version of the ADBS with respect to the a priori defined optimization criteria. The paper provides a customizable R syntax for building reliable, valid, and theoretically well-founded short scales.

**Supplementary Information:**

The online version contains supplementary material available at 10.1038/s41598-025-12087-3.

## Introduction

Assessing data with self-report questionnaires happens between the conflicting priorities of maximizing reliability and validity and keeping the burden for respondents as low as possible^1^. Lengthy measurements require time and financial resources, while leading to less acceptance, careless responding, respondent fatigue and lower participation rates^2,3^. Particularly in the context of health and prevention^4^ it is important to keep assessments scarce in order to make them practical and avoid nonresponse. Thus, there is an apparent need for efficient and psychometrically sound short scales.

Self-report questionnaires have often been shortened by choosing items with the highest item-total correlation or the highest loadings in exploratory factor analysis^1^. This approach entails a trade-off between reliability and validity^5^. Relying on statistical information such as factor loadings, error variances and internal consistency may alter the dimensionality or factor structure, hence affecting a short scale’s construct validity^6^. What is more, the stepwise procedure, in which few or even a single statistical value determines the in- or exclusion of items sequentially, may overlook important item combinations^7^. With increasing awareness of the shortcomings of the traditional factor analytic approach^8,9^ more sophisticated methods such as automatic optimization algorithms or meta-heuristics have been applied^10^ one of those being the Ant Colony Optimization (ACO) algorithm^6^.

The ACO principle is derived from the behavior of an ant colony trying to find and establish the shortest path between their nest and a source of food^11^. Experiments showed that ants leave chemical traces in the form of pheromones on their routes. As more ants are able to reach the food in less time on the shortest route, the pheromones accumulate on that particular path, again attracting other ants until the majority of the colony chooses this route. This principle has been transferred to short scale construction^12^. As different ants randomly find paths between nest and food, different subsets of items are randomly drawn from an available item pool in the initial phase. Research has to define optimization criteria such as model fit, factor saturation, or relationships to external variables (analogous to the shortest route for ants) that are used to assign pheromones to those items that best meet the optimization criteria. These pheromones then increase the probability that the items are selected again in the following draws. With more and more iterations, the algorithm reliably yields a high-quality solution for a short scale, just as the ant colony reliably establishes the optimal route between nest and food. However, due to its heuristic nature the ACO algorithm does not test all possible solutions from a given item pool, potentially resulting in different optimal solutions in separate runs^6^.

Constructing short scales using meta-heuristics such as the ACO algorithm has been successfully established in personality^8,13,14^ and educational research^10,15^. Different adaptations of the ant colony optimization have been shown to be feasible and promising alternatives to traditional item selection strategies^10,16^. To our knowledge, these methods are only slowly migrating to the health sciences, ostensibly due to very technical descriptions in scientific publications and a lack of comprehensible and applicable instructions for the re-use by other researchers. This contrasts with the great need for short scales in the prevention of common diseases through health behavior change.

In practice, short scales are typically used to screen for mental disorders such as depression^17^ and health risk behaviors such as alcohol consumption^18^ and physical inactivity^19^. In the tradition of brief intervention approaches, motivational constructs such as self-efficacy are often assessed with brief measures to provide tailored feedback^20,21^. According to the Transtheoretical Model of Behavior Change^22^weighing the pros and cons of changing one’s behavior is a central motivational process in the transition through the stages of change from precontemplation to action. This so-called decisional balance was frequently assessed and used for individualized feedback in brief interventions targeting alcohol use in different populations^23–26^. An exhaustive assessment of all potential advantages and disadvantages of cutting down drinking is time-consuming, complex and may compromise people’s readiness to stay engaged with an intervention. Thus, the measurement of decisional balance is a perfect example where reliable and valid short scales are needed.

The purpose of this paper was to show the practical implementation of the ACO algorithm by constructing a reliable and valid short version of the German Alcohol Decisional Balance Scale^27^. We aimed to compare the short scale produced by the algorithm with the internationally well-established brief version of the same instrument^28^ as well as an item selection based on the highest factor loadings. By doing this in a readily accessible way and by providing customizable R syntax, we hope to support the dissemination of meta-heuristics such as the ACO algorithm in short scale construction projects in the health and prevention sciences.

## Methods

### Sample

For this paper, three subsamples from studies conducted by the research network EARLy INTerventions in health risk behaviors (EARLINT) were merged. We included participants with at-risk alcohol use who completed the Alcohol Decisional Balance Scale (ADBS), resulting in an overall sample of *N* = 1,834 aged between 18 and 67 (*M* = 41.2, *SD* = 12.8), including 348 women (18.9%). All procedures contributing to this work comply with the Helsinki Declaration of 1975, as revised in 2013. The Ethics Committee of the University Medicine Greifswald has approved the original studies. All participants gave their informed consent to participate in the respective studies. The respective samples are described below:

*Study 1: Transitions in Alcohol Consumption and Smoking (TACOS).* The data were collected partly as paper pencil surveys and partly through computer-assisted personal interviews during the TACOS project in 1996/97^29–31^. TACOS was a longitudinal observation study aiming to examine data from the general population in Northern Germany on smoking and alcohol usage. Study participants were randomly drawn from a population sample alcohol and tobacco users in the northern German city of Luebeck. A subsample of at-risk alcohol users from wave 2 (30 months after baseline) was used because the full ADBS was not administered until then. Surveys for this assessment were conducted by self-administered paper-pencil questionnaires. Additionally, non-respondents were contacted with reminders via mailings, telephone calls or personal visits, resulting in a response rate of 86.1%^32^. Of 518 baseline participants, 310 provided data on the ADBS and were used in our analyses.

*Study 2: Implementing Early Intervention for Alcohol Misuse in the General Hospital (KIK).* This randomized controlled trial aimed to investigate the comparative efficacy of brief alcohol interventions delivered by routine care hospital physicians versus by a liasional service. For this purpose, patients were recruited from four general hospitals in North-Eastern Germany between 2002 and 2004^33,34^. Patients between 18 and 64 years were proactively approached by study staff and asked for their consent for an alcohol screening. After an initial screening of 14,332 patients, a total of 2,337 participants with at-risk alcohol use agreed to a subsequent diagnostic interview. Of these, 1,281 patients were eligible for trial inclusion, and *n* = 1,166 agreed to participate in the KIK study, providing written informed consent. A total of *n* = 771 provided complete data on the ADBS, and were used for the following analyses.

*Study 3: Randomized Controlled Trial of an Expert System for Patients with at-risk Drinking*,* Alcohol Abuse and Alcohol Dependence in General Hospital (EXTRA)*. This randomized controlled trial tested the efficacy of a computerized expert system intervention for unhealthy alcohol use compared to an untreated control group. Non-dependent medical inpatients from the general hospital of Luebeck were proactively recruited between 2004 and 2005^35^. A health screening survey was completed by 2,949 patients aged between 18 and 64, who were then invited to give their informed consent to participate in the EXTRA trial. Of 954 study participants with at-risk alcohol use from both treatment arms, 753 completed subsequent standardized interviews within 24 h after the screening, including the ADBS, and were included in our analysis sample.

### Measure

Data for the item selection was used from the German *Alcohol Decisional Balance Scale* (ADBS, Hannöver et al., 2003). The original version consists of 20 items that can be assigned to 2 factors: pros and cons of drinking^27^. Additionally, 6 items (3 pros and 3 cons) were added to assess aspects regarding overall health and fitness for at-risk consumers. Participants were asked to rate the importance of each item in their decision to consume alcohol or not. The items of the full questionnaire are presented in Table 1. A 5-point Likert scale (1 = not at all important; 2 = not very important; 3 = somewhat important; 4 = very important; 5 = extremely important) was used to answer the items. Although the two-factor structure of the questionnaire is not universally supported^36^ it is still predominantly applied to the questionnaire^37^. We decided to keep this factor structure for the item selection for better comparability with previous work and consistency within the theoretical framework of the TTM. The 10-item short scale was constructed by choosing five items for each factor based on item difficulty and item-total correlation^36^.

**Table 1:** Factor loadings for the full item pool, the original short scale, the ACO-P scale, the ACO-C scale, and the factor loading scale.

#	Sub.	Item text	Full item pool (26 items)	Original short scale (10 items)	ACO-*P* scale (10 items)	ACO-C scale (11 items)	Factor loading scale (10 items)
1	Pros	Drinking helps me to loosen up and express myself.	0.83	0.85	-	-	0.85
2	Pros	I like myself better when I am drinking.	0.77	-	0.77	0.79	-
3	Cons	Because I continue to drink, some people think I lack the character to quit.	0.79	-	-	-	-
4	Pros	Drinking helps me to deal with my problems.	0.86	-	-	-	-
5	Cons	Having to lie to others about my drinking bothers me.	0.82	-	-	-	-
6	Cons	Some people try to avoid me when I drink.	0.61	-	-	-	0.85
7	Pros	Drinking helps me to have fun and socialize.	0.83	0.92	-	0.75	0.89
8	Cons	Drinking interferes with my functioning at home and/or at work.	0.85	-	-	0.68	-
9	Pros	Drinking makes me more of a fun person.	0.71	0.86	-	-	0.84
10	Cons	Some people close to me are disappointed in me because of my drinking.	0.76	-	0.85	0.88	0.89
11	Cons	My drinking causes problems with others.	0.70	-	0.97	0.97	0.96
12	Cons	I seem to get into trouble when drinking.	0.25	-	0.93	0.92	0.92
13	Cons	I could accidently hurt someone because of my drinking.	0.31	-	0.74	-	-
14	Pros	Not drinking at a social gathering would make me feel too different.	0.74	0.56	-	-	-
15	Cons	I am losing the trust and respect of my coworkers and/or spouse because of my drinking.	0.78	0.64	-	0.82	0.82
16	Pros	Drinking helps me to give me energy and keeps me going.	0.86	-	0.86	-	0.79
17	Pros	I am surer of myself when I am drinking.	0.69	-	0.87	0.87	0.85
18	Cons	I am setting a bad example for others with my drinking.	0.88	0.74	-	-	-
19	Pros	Without alcohol, my life would be dull and boring.	0.94	-	-	0.83	-
20	Pros	People seem to like me better when I am drinking.	0.92	-	0.73	0.80	-
21	Cons	Drinking causes me to gain weight.	0.74	0.72	-	-	-
22	Pros	Drinking helps me to relax.	0.82	0.49	0.68	-	-
23	Pros	I like to taste of alcohol drinks.	0.70	-	-	-	-
24	Cons	Alcohol is bad for my health.	0.43	0.70	0.47	0.49	-
25	Cons	When I drink alcohol, I am physically less fit.	0.55	0.71	-	-	-
26	Pros	A certain amount of alcohol is good for my health.	0.56	-	-	-	-

### Data analysis

The ACO algorithm was based on previous works of Leite et al.^6^Olaru et al.^8^ and Janssen et al.^13^. The code was written in R^38^ and is freely accessible in Supplement A as a function and allows for easy modification of parameters and specifications of the algorithm, depending on the individual requirements of the research question.

The basis for the model estimation was a two-factor Confirmatory Factor Analysis (CFA) which was implemented explicitly into the code, using the lavaan package^39^. The CFA was estimated with the Weighted Least Squares Mean and Variance adjusted (WLSMV) estimator. The two-factor structure is well-established and theoretically important for the instrument. We restricted the algorithm to select items for each factor only from those that had been originally assigned to that factor. The algorithm requires an a priori defined length of the short scale, i.e. how many items should be selected from the item pool. We selected 5 items per factor (pros and cons), in line with the well-established existing short-scale for the ADBS^36^. The overall pheromone (φ) value was defined as a sum function of pheromone values from the individual optimization criteria. For the evaluation of the model fit, we included the Comparative Fit Index (CFI) and the Root Mean Square Error of Approximation (RMSEA). The optimization criteria were met if the CFI was ≥ 0.96 and the RMSEA was ≤ 0.06:

1 $$\:{\phi\:}_{CFI}=\frac{1}{1+{e}^{95-100CFI}}$$

2 $$\:{\phi\:}_{RMSEA}=1-\frac{1}{1+{e}^{5-100RMSEA}}$$

They were integrated equally weighted to estimate the pheromone of the model fit:

3 $$\:{\phi\:}_{Fit}=\frac{{\phi\:}_{CFI}+{\phi\:}_{RMSEA}}{2}$$

Additionally, McDonald’s ω was introduced as a reliability coefficient for the estimation of the pheromone level. The optimization criterion was set at ω ≥ 0.9:

4 $$\:\omega \: = \:\frac{{\left( {\sum \: _{{i = 1}}^{n} \lambda \:_{i} } \right)^{2} }}{{\left( {\sum \: _{{i = 1}}^{n} \lambda \:_{i} } \right)^{2} + \:\sum \: _{{i = 1}}^{n} 1 - \:\lambda \:_{i}^{2} \:}}$$

5 $$\:{\phi\:}_{Rel}=\frac{1}{1+{e}^{9-10\omega\:}}$$

Because the full scale of the ADBS is considered a well-established, reliable and valid instrument, we aimed to select items that would reflect the scale as closely as possible. Therefore, we chose the correlation between the original scale and the new short scale as the third optimization criterion. The correlation should be at least 0.85:

6 $$\:{\phi\:}_{Corr}=\frac{1}{1+{e}^{85-100cor}}$$

And finally, the difference of the factor correlation between the original scale and the short scale was added as the fourth optimization criterion. This criterion was chosen to ensure that the relations between the subscales of the short form would reflect their intended relation, as measured by the full scale, as closely as possible. The difference should stay below 0.03 to ensure that the latent dimensions of the short scale are associated with each other in a comparable way to the original scale:

7 $$\:{\phi\:}_{Fc}=1-\frac{1}{1+{e}^{3-100max}}$$

In addition to the optimization criteria, the ACO algorithm requires three more parameters to be set a priori: the number of ants, the number of iterations, and the evaporation rate. These parameters require careful consideration and testing to balance consistency, sufficient exploration, and the run time of the algorithm. Depending on computational power, sample size, and complexity of the problem, the run time might vary from seconds to hours. Reducing the exploration to shorten run times might lead to overlooking better solutions. To our knowledge, there are no general standards to define these parameters. Rather, they are usually chosen on case-by-case basis, depending on the specifications of the data and the algorithm itself.

The number of ants determines the number of item combinations examined per iteration. More ants ensure more exploration and typically result in higher quality solutions. However, a higher number of ants also increases the run time of the algorithm. The pheromone did not show substantial increase after about 30 ants. However, the variance of optimal solutions still decreased with more ants until about 70–80 ants. To examine this in more detail, the optimal solutions were further examined in regard to the individual optimization criteria. The results showed that the optimal solutions found by the algorithm comfortably met the cut-off points of the optimization criteria for every configuration. The best solution found by the algorithm was the same for all configurations with at least 40 ants. Increasing the number of ants resulted in more homogenous findings across different runs, notably so for 60 ants or more.

The adequate number of iterations for the algorithm depends on the problem size and how much exploration is needed to find optimal solutions. More exploration would benefit from more iterations to avoid convergence before the optimal solution is found. If the problem is more easily assessable and less exploration is required to find an optimal solution, fewer iterations might be sufficient while a high number of iterations might unnecessarily increase the runtime without increasing the quality of the solutions. For the selection of items from the ADBS, the ACO algorithm found good solutions even with low numbers of iterations. There was only a slight increase in quality at higher numbers, which would not necessarily justify higher runtime. However, the algorithm consistently found the overall best solution across several runs in a range of 30 to 90 iterations.

The evaporation rate determines how slow previous solutions lose influence for later iterations. A higher value for the evaporation rate means that the pheromone evaporates more slowly. This results in fewer numbers of significant iterations and thus less diversity in the solutions found by the algorithm. This may help to produce more consistent results but it also might potentially lead to better solutions being overlooked. The results for varying evaporation rates showed similar quality for the best solution found by the algorithm with only marginal increases for the model fit indices around a rate of 0.8 to 0.9. Overall, the algorithm showed more homogenous item selections for evaporation rates around 0.5 which did not change with increasing values. Thus, higher values of the evaporation rate, that would cause longer runtimes, were not necessary for consistent solutions.

Once the initial parameters (number of selected items, number of ants, evaporation rate, optimization criteria, estimation of pheromone level, estimation of pheromone update) are fixed, the algorithm starts through several iterations until the convergence criterion is met. For the first run, the pheromone level is equal across all items, resulting in the pseudo-random selection for the first variations. The selected item sets are evaluated based on the optimization criteria and the pheromone level $\:{\phi\:}_{1}$ is estimated for all estimated models. The best-so-far solution can then be determined (iteration 1) or the best pheromone level of the current item selections can be compared to the best-so-far pheromone from previous iterations. If the current item set has a higher pheromone value, the best-so-far pheromone is updated. The pheromone weight is added to the initial pheromone. Then the algorithm repeats everything starting from the item selection until the convergence criterion is met and a final item selection is found.

To examine the consistency of the solutions generated by the algorithm with varying parameters, each variation was run five times, resulting in five optimal solutions for each variation. The results were evaluated in regards to the respective optimization criteria, i.e., model fit (CFI, RMSEA), internal consistency (McDonald’s ω), scale correlation, difference in factor correlation, as well as their factor loadings. Two approaches were chosen to compare the ACO solutions against the established scales and the traditional item selection of choosing items with the highest factor loadings: (1) The best solution, according to the highest pheromone level, was selected from the five possible optimal solutions (ACO-P scale); (2) a consensus scale was selected by choosing the all items that were selected in at least 60% of the optimal solutions (ACO-C scale). Both of these selections were then compared to the full scale of the ADBS, the established ADBS short scale, and a selection of 10 items based on the highest factor loadings in regard to their psychometric characteristics. The aim was to figure out whether the items selected by the ACO algorithm are comparable or better than the existing scales or the traditional selection strategy of choosing items with the highest factor loadings.

## Results

### Factor structure of the original full and short scale

To account for the different settings and population samples used in the following analyses, we conducted a preliminary measurement invariance analysis across the three subsamples. The results showed no substantial decline of model fit, indicating scalar measurement invariance. Additionally, due to the severe skew in the gender ratio, we conducted measurement invariance testing between male and female participants, which also indicated scalar invariance (see Supplement B). Therefore, the three subsamples were analyzed jointly.

First, a two-factor CFA was estimated with all 26 items. Overall, the model showed a good fit (see Table 2), although the RMSEA is higher than the conventional threshold for a good fit (*χ²* (298) = 2507.53, *p* < .001, CFI = 0.953, RMSEA = 0.072). However, it should be noted that the application of conventional thresholds has been frequently criticized for models with categorical data and should be interpreted cautiously^40,41^. Overall, factor loadings ranged from *λ* = 0.25 to *λ* = 0.94 with an average of *λ* = 0.72 (see Table 1) and a correlation of 0.56 between both latent factors. McDonald’s omega indicated high internal consistency with similar values for both factors (*ω*_*pros*_ = 0.93; *ω*_*cons*_ = 0.94).

**Table 2:** Fit and optimization criteria for the full item pool, the original short scale, the ACO-P scale, the ACO-C scale, and the factor loading scale.

	Χ^2^ (df)	CFI	RMSEA	Omega		Factor loadings			Factor correlation
				Pros	Cons	Mean	Min.	Max.	
Full item pool	2507.53 (298)	0.953	0.072	0.93	0.94	0.72	0.25	0.94	0.56
Original short scale	366.19 (34)	0.975	0.080	0.88	0.81	0.72	0.50	0.92	0.42
ACO-P scale	111.07 (34)	0.996	0.039	0.89	0.90	0.79	0.47	0.97	0.56
ACO-C scale	165.12 (43)	0.995	0.044	0.89	0.92	0.79	0.48	0.96	0.56
Factor loading scale	599.08 (34)	0.981	0.104	0.93	0.95	0.87	0.79	0.96	0.51

All *Χ²*-tests are statistically significant at *p* < .001. *CFI * Comparative fit index, *RMSEA * Root mean square error of approximation, *Omega* McDonald’s Omega.

The original 10-item short scale showed an overall substantial improvement in model fit, with the exception of the RMSEA, (*χ²* (34) = 366.19, *p* < .001, CFI = 0.975, RMSEA = 0.080). The factor correlation decreased substantially to 0.42 The range of the factor loadings narrowed down due to items with lower loadings not being selected for the short form (now *λ* = 0.50 to *λ* = 0.92), the average remained at *λ* = 0.72. Compared to the full item pool, McDonald’s omega decreased, especially for the cons factor (*ω*_*pros*_ = 0.88; *ω*_*cons*_ = 0.81).

Another selection of 10 items was based on factor loadings. We selected 5 items with the highest loadings for each factor, respectively. This resulted in a comparable CFI to the original 10-item short scale (*χ²* (34) = 599.08, *p* < .001, CFI = 0.981), but a substantially worse value for the RMSEA (RMSEA = 0.10). The factor loadings were predictably higher and within a narrower range (*λ* = 0.79 to *λ* = 0.96). Consequently, the selection showed higher values for McDonald’s omega than the original 10-item scale (*ω*_*pros*_ = 0.93; *ω*_*cons*_ = 0.95).

### ACO algorithm results

**Fig. 1:**
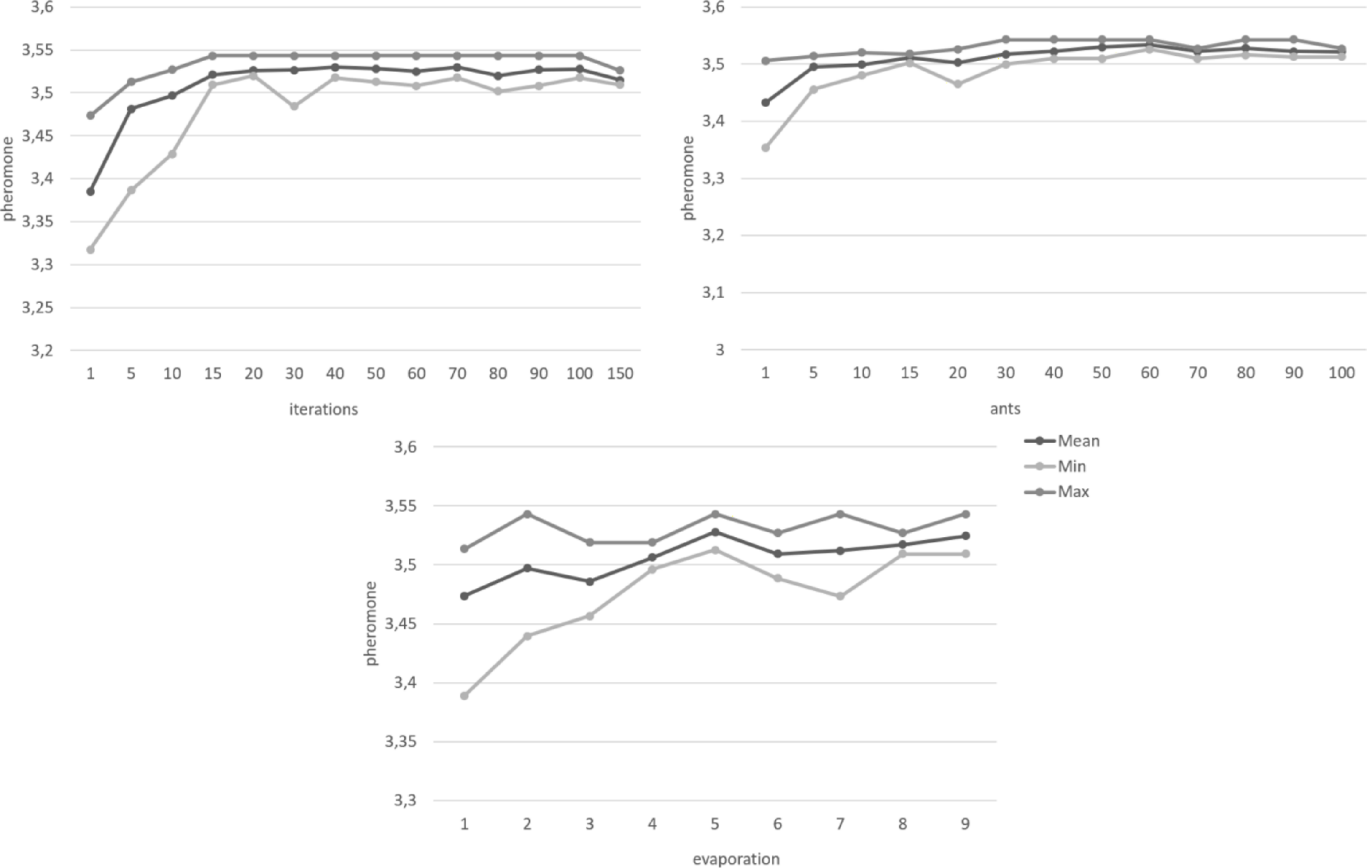
Changes in the overall pheromone depending on the number of iterations, ants, and the evaporation rate.

**Fig. 2:**
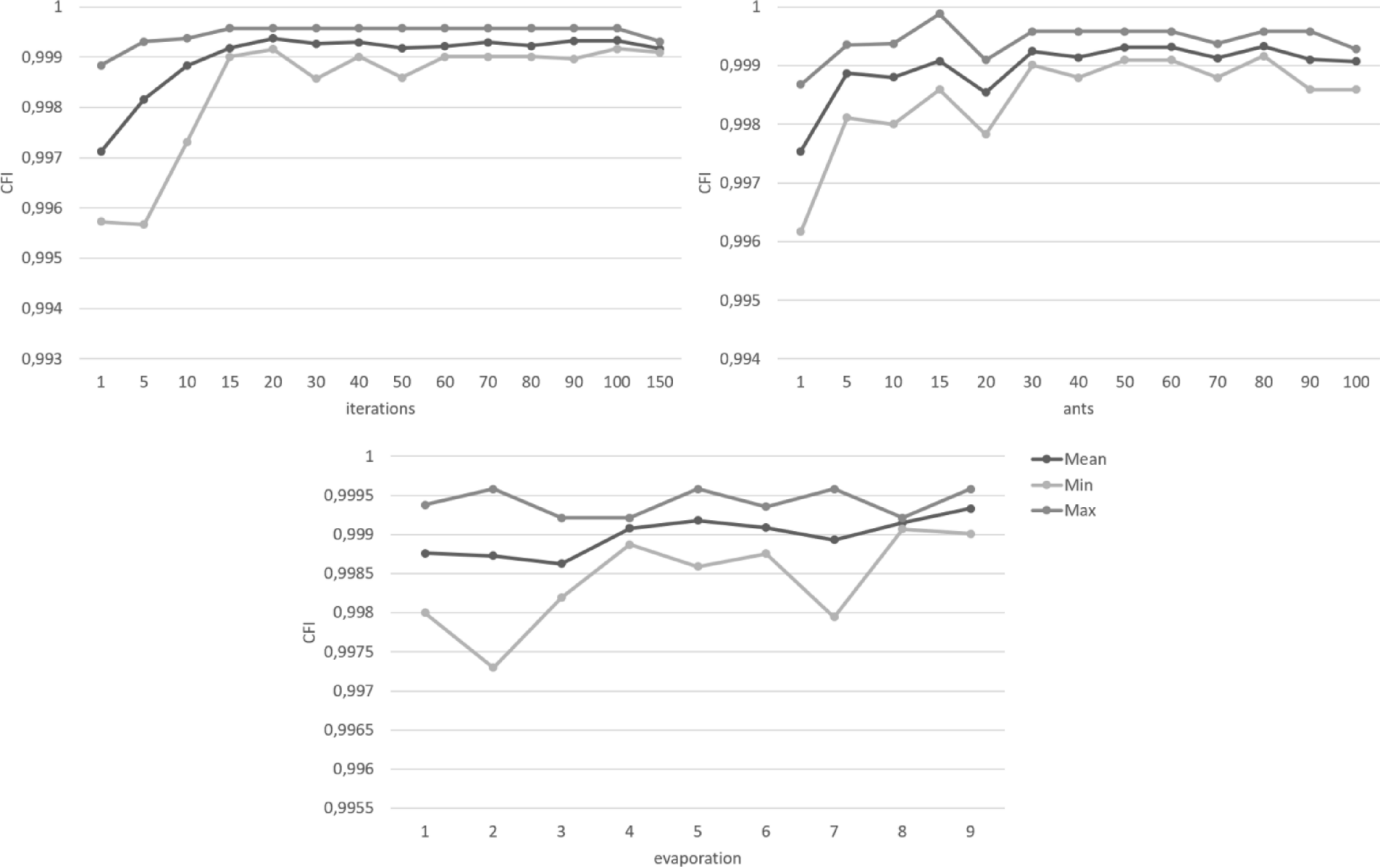
Changes in the comparative fit index (CFI) depending on the number of iterations, ants, and the evaporation rate.

**Fig. 3:**
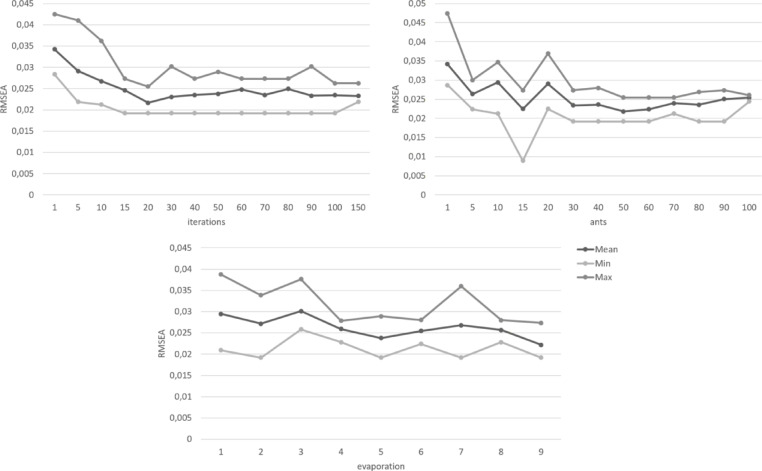
Changes in the root mean square error of approximation (RMSEA) depending on the number of iterations, ants, and the evaporation rate.

**Fig. 4:**
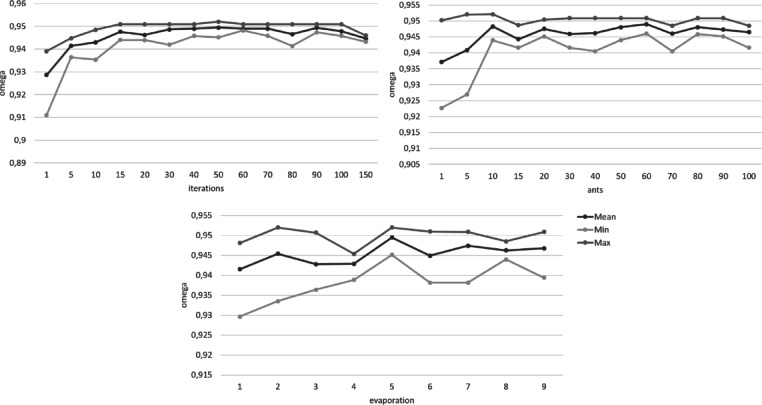
Changes in the McDonald’s omega depending on the number of iterations, ants, and the evaporation rate.

**Fig. 5:**
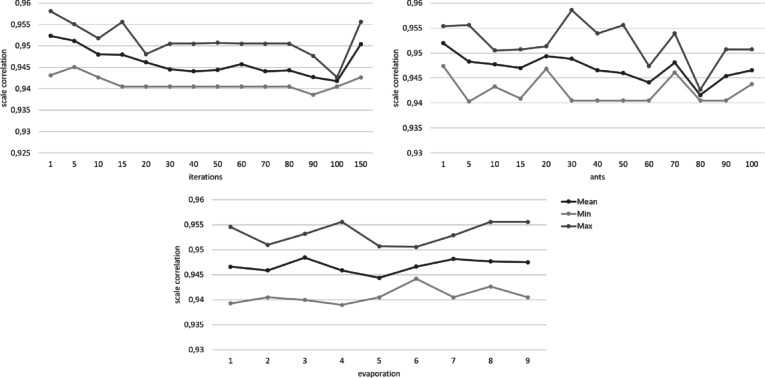
Changes in the correlation between the original scale and the ACO short scale depending on the number of iterations, ants, and the evaporation rate.

**Fig. 6:**
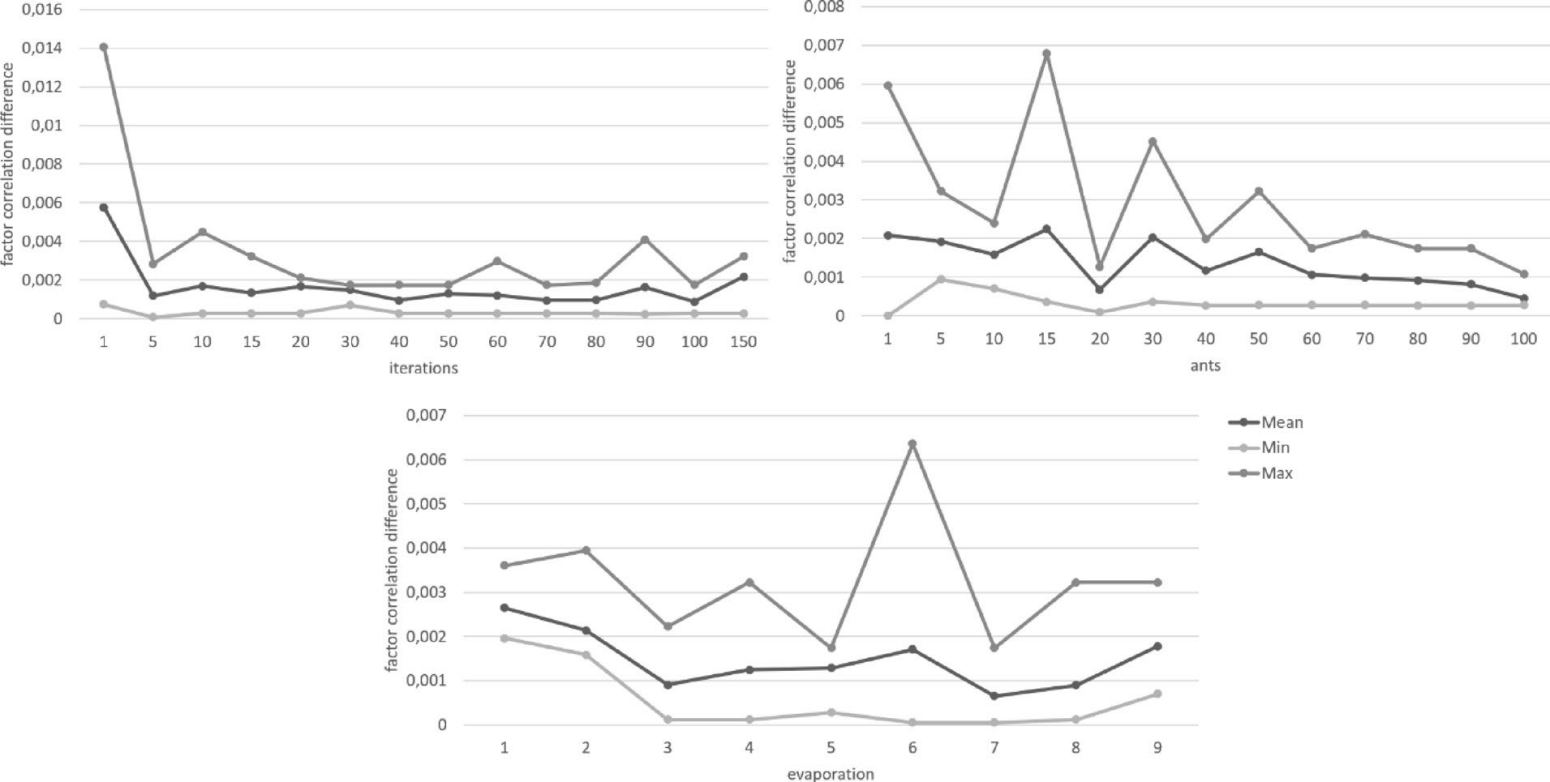
Changes in the difference of factor correlation between the original scale and the ACO short scale depending on the number of iterations, ants, and the evaporation rate.

Figures 1, 2, 3, 4, 5 and 6 show the overall pheromone and the individual optimization criteria for the optimal solutions found with varying values for ants, evaporation rate and iterations as described in the data analysis section. After completing five runs, the ACO algorithm found three unique solutions as the “optimal solution”. For the ACO-P scale, we selected the solution with the overall highest pheromone. This specific item combination was determined as optimal in two of five runs. The model fit showed a substantial improvement to both the full item pool and the original short scale, with all optimization criteria reaching their cut-off points (*χ²* (34) = 111.07, *p* < .001, CFI = 0.996, RMSEA = 0.039; *ω*_*pros*_ = 0.89; *ω*_*cons*_ = 0.90) and a factor correlation of 0.56.

Of the 10 items selected for the solution with the highest pheromone, 8 were also selected in at least three of the other runs and 3 items were consistently selected in every run, thus showing a substantial overlap in the final item selection. These 11 items were used for our second approach as the ACO-C scale (consensus scale=. The model fit was almost identical to the ACO-P scale due to their similarity in item selection (*χ²* (34) = 165.12, *p* < .001, CFI = 0.995, RMSEA = 0.044; *ω*_*pros*_ = 0.89; *ω*_*cons*_ = 0.92).

## Discussion

Using the ACO algorithm, we used two approaches to select items for valid and reliable 10- and 11-item short versions of the German ADBS, a self-report questionnaire measuring the perceived importance of different aspects in people’s decisions to drink alcohol. The new short versions showed a better fit to the data than the original 10-item short scale as well as a selection based on the highest factor loadings and included eight items from the available item pool that were not present in the original short version of the ADBS. Thus, the short scales differed not only in their psychometric properties, but also in the content representing the pros and cons of alcohol use. Our study demonstrated that the ACO algorithm is able to outperform traditional methods of short scale construction in terms of psychometric properties. The selection of items with the highest factor loadings resulted in a 10-item short form with a fit comparable to the established short form. However, only 4 items overlapped with the original short form. Overall, this seems to indicate that short scale development approaches based on a single criterion can result in widely diverging selections. However, the fit indices were comparable to the original short scale, which is good for the CFI and more questionable for the RMSEA. Overall, both ACO selections yielded superior model fit.

However, our present analyses focused only on the optimization of psychometric properties such as model fit indices and factor correlation. While the results clearly favor both ACO short scales, it may be equally important to consider theoretical aspect, such as the prediction of behavior change or correlations with related constructs, in determining the performance of a short scale.

### The ACO algorithm in research practice

Inspired by the behavior of ants foraging for food, the ACO algorithm searches an available pool of items to find the optimal solution to a brief questionnaire^11,12^. To do so, the algorithm repeatedly selects subsamples of items and attempts to maximize a priori defined criteria that the resulting short scale should satisfy. The ACO algorithm has a major strength in its ability to consider multiple optimization criteria simultaneously, which sets it apart from other approaches to short scale construction, such as confirmatory factor analysis. However, meta-heuristics in general, and the ACO algorithm in particular, have so far only been used very rarely in practice, most frequently in personality^8,13,14^ and educational research^10,15^. The ACO algorithm used for our analyses was implemented in R (see Supplement A), a programming language familiar to most researchers. By doing this, we aim to overcome at least one barrier (e.g. Python knowledge) that may prevent the ACO algorithm from migrating into the consciousness and toolbox of those who create short scales.

Configuring the ACO algorithm involves deciding on various optimization criteria and their cut-off values, as well as choosing the number of ants, iterations, and the evaporation rate. To accomplish this task, a sufficiently precise definition of the research question is necessary, including the nature of the underlying theoretical construct to be measured and its relationship to other important variables. At the same time, the configuration of the ACO algorithm offers an opportunity to secure the quality of short scale construction practice. By mandating researchers to form hypotheses before analyzing data and shortening existing questionnaires, potentially questionable research practices such as HARKing^42^ are prevented by design. Meta-heuristics are a powerful tool for building reliable and valid short scales. However, it is important to note that their output is dependent on the quality of the data given to the algorithm. The ACO algorithm cannot compensate for poor data quality, a problem commonly and colloquially referred to as “garbage in - garbage out”^43^.

The optimization criteria used by the ACO algorithm can be manifold. This paper’s example included psychometric model fit criteria CFI, RMSEA, and McDonald’s Omega. The correlation between the two latent factors, the pros and cons of alcohol use, was aimed to be as close as possible to the corresponding correlation within the original (long) scale to ensure equivalence between the two questionnaires. Other optimization criteria may be considered depending on the area of application and the available data. Previous studies have used model fit indices like CFI and RMSEA^6,13^ but occasionally also integrated other optimization criteria such as test-retest reliability and correlations with external variables^44^ and discrimination performance^10^.

Two approaches for the implementation of the ACO algorithm were illustrated. First, items were selected based on what the algorithm identified as the best solution over multiple runs according to the highest pheromone value (the ACO-P scale). This approach can be especially helpful for comparisons with other methods and with items from well-established constructs with a strong theoretical and empirical foundation. It should ideally be used in scenarios, where a high overlap across multiple runs is either very likely or unnecessary. However, for a practical application of the algorithm in clinical and preventive settings, we believe the second approach (the ACO-C scale) could produce more reliable results. The advantage of this approach is that the researcher can be sure that the chosen items were reliably selected multiple times by the algorithm, resulting in a more stable solution. This can be even more effective if a higher selection rate is chosen for the final item selection. Especially in situations with larger item pools of more varied validity and reliability, the ACO algorithm could serve as an effective tool to preselect items over multiple runs and based on multiple empirical criteria, followed by another selection based on more theoretically focused validity assessments. This could limit the effect of the pseudo-randomization and increase replicability of the results.

### Comparison of the original ADBS and the ACO short scales

The primary distinction between the original ADBS short scale^28^ and the both brief versions developed by the ACO algorithm in this paper is the content covered by the items. The original short scale shared only two (ACO-P scale) or three (ACO-C scale) items respectively with the scales selected by the ACO algorithm (cf. Table 1), which address the relaxing (item 22) and health-threatening effects of consuming alcohol (item 24) for the ACO-P scale and the original short scale, respectively. The latter was also selected for the ACO-C scale which could point to health concerns being one of the most consistently relevant items of the ADBS. The original short scale asks respondents to rate the extent to which their decision to drink is influenced by their role in social situations, for example being sociable (items 1 and 14) or feeling like the odd one out when not drinking (item 14). In contrast, the ACO short scales places greater emphasis on self-evaluation (items 2 and 17) and the evaluation by others (items 10 and 20). The ACO short scales appear to address more serious consequences that may result from hazardous alcohol consumption such as causing problems with others (item 11) or as getting into trouble (item 12), compared with more general health consequences that may already be experienced by people with lower but still at-risk consumption such as gaining weight (item 21) or feeling less fit (item 25) in the original short scale. The variations in item selection may be due to the methods or study samples used to construct the respective short scales. Different advantages and disadvantages of alcohol consumption may be important for people’s decisional balance, depending on their problem severity and consumption pattern. Consequences of alcohol consumption related to hazardous drinking, such as those measured by the ACO short scales, may have a particular action-guiding effect for drinkers who have not yet experienced them.

However, it is also possible that the algorithm selected items closer related to hazardous drinking and alcohol use disorders because these items were more heavily represented in the original scale. The ACO algorithm was supposed to find items that would reflect the original scale as closely as possible as indicated by the scale correlation and factor correlation. This could have led to a stronger representation of items more closely associated with alcohol use disorder rather than the six items that were added to represent more general health concerns. Contrary, items for the original ADBS short scale were selected specifically with the aim of representing people with low alcohol consumption^36^. In any case, the varying item content may have implications for preventive efforts that address people’s decisional balance regarding their alcohol consumption. These interventions^23–26^ often involve individualized feedback based on responses to questionnaires like the ADBS. Thus, changing questionnaire content may require adapting intervention content accordingly.

From a practical standpoint, differences in item selection highlight the importance of aligning item content with specific population and intervention goals. In clinical settings, a focus on more severe consequences of alcohol use may be beneficial for addressing attitudinal shifts, particularly for individuals with increased vulnerability for alcohol use disorders. For public health efforts targeting the general population and all alcohol users, emphasizing the everyday impacts of alcohol use on social functioning or general well-being may be more effective. Given the fact that different combinations of items may compete for the best brief questionnaire measuring alcohol-related decisional balance or any other construct, a tailored approach to short scale selection may be indicated, considering the specific aim and purpose of each scale. As such, the ACO algorithm may be a valuable tool in the multi-step procedure of constructing reliable and valid short scales. If the future scope and aim of a short scale can be translated into and quantified as optimization criteria, the ACO algorithm will be able to consider these criteria simultaneously, in addition to the traditional psychometric benchmarks. However, further construction steps such as a formal content validity assessment or cross-validation may be necessary and outside of the scope of the ACO algorithm.

### Strengths and limitations

To the best of our knowledge, this study is one of the first efforts to implement the ACO algorithm entirely in R. The customizable syntax (provided in Supplement A) facilitates the use of meta-heuristics in short scale construction. However, it should be noted that the determination of parameters for the algorithm (number of ants, evaporation rate, number of iterations) as well as the threshold for the ACO-C scale in this study rely on a purely empirical approach and future studies should be mindful about their specifications. Simulations studies may be warranted for a reliable and more generalizable determination of these parameters. Furthermore, the RMSEA values especially for the comparison of our models should be interpreted with caution due to both the ordered categorical data used in our models^41^ and the large sample and high number of *df* which can mask misspecifications^45^ due to small differences between the RMSEA values.

Based on a large sample of at-risk drinkers, the merits of the ACO algorithm are to provide a short version of an established questionnaire measuring alcohol decisional balance. The short scales were compared on the basis of their psychometric performance. It should be noted that, although not part of our analyses, theoretical aspects such as validating the predictions of the Transtheoretical Model and definition of the target population are often at least as important in determining the quality of a short scale. The marked differences in content between the original 10-item short scale and both ACO short scales may indicate possible different applications or target groups for interventions. This could be of interest for future research.

The results found by the ACO algorithm in this study might be limited in their generalizability. The samples for the EARLINT research consortium that were the basis of the current analysis were specifically recruited for studies in Germany and thus may not generalize cross-culturally or to samples with lower or higher alcohol consumption. Furthermore, the current study was unable to perform a thorough examination of the content validity of the selected items which would be crucial to ensure an adequate alignment to the theoretical framework of the ADBS scale. It would be optimal to conduct further formal assessments to ensure that the pros and cons of decisional balance are adequately conceptualized in the short scale resulting from the ACO algorithm. Due to the lack of external criteria we could use to account for validity, this served as the closest solution to keep the latent construct of decisional balance as close to the original intention as possible. This would be a more relevant concern for item pools that are not as well-established and anchored in a theoretical framework as the items of the ADBS scale. Furthermore, due to time and resource restrictions we were unable to cross-validate our results with an independent sample. This should be included in future analyses to ensure replicable results. However, the aim of this paper was not to find a universally applicable ADBS short scale but rather to use the ADBS scale as an example to demonstrate the ACO algorithm as an efficient tool for item selection in health and prevention science.

### Outlook

The ACO algorithm has shown itself to be effective in selecting a well-performing short scale in a reproducible and consistent way across several runs. Because the algorithm can be individually adjusted to specific research needs, it is a promising tool to facilitate individualized tailoring of surveys even with complex requirements due to the ability to include a variety of optimization criteria. The algorithm can be used to reduce large item pools while considering multiple, possibly complex, criteria simultaneously, including theoretical considerations, such as taking the factor structure into account or by including relations to covariates. Furthermore, individually tailored interventions have proven to be effective tools in population-based prevention research^46–49^ Meta-heuristic algorithms such as the ACO are helpful and reliable methods to further improve this individualization. Health assessment and treatment has increasingly started to integrate more technological approaches through e-health, m-health, and artificial intelligence^50^. However, it should also be noted that a successful implementation requires balancing between the technological aspects, ethical considerations, and most importantly the well-being of the patients. While machine learning can be such a helpful tool for selecting item and optimizing assessments to be more individualized and time efficient, it is crucial to ensure that the result is still theoretically sound and thus suitable for the intended intervention. Thus, future studies using such algorithms should further take care to account for formal content and predictive validity assessments and cross-validations with independent subsamples to ensure clinical applicability and generalizability of the new scales.

## Electronic supplementary material

Below is the link to the electronic supplementary material.

Supplementary Material 1

Supplementary Material 2

## Data Availability

The datasets generated and/or analysed during the current study are not publicly available due to agreements made in the informed consent but are available from the corresponding author on reasonable request. The annotated R script of the ACO algorithm is available as Supplement A. This study and the analysis plan have not been preregistered.
