# Supplementary material for: Application of the ant colony optimization algorithm for the construction of a short version of the German alcohol decisional balance scale
Source: Sci Rep. 2025 Jul 25;15:27122. doi: 10.1038/s41598-025-12087-3 (PMC12297269; doi:10.1038/s41598-025-12087-3)
Supplement: Supplementary file 1 — Supplementary Material 1 [file 41598_2025_12087_MOESM1_ESM.html]

Constructing a short version of the German ADBS: Application of the ACO algorithm


# Constructing a short version of the German ADBS: Application of the ACO algorithm

#### 2024-06-27

### 1. read data

depending on your file / program you might not need the conversion to
numeric

```
library(readstata13) # read .dta from Stata
data <- read.dta13('E:/ACO/Daten/ADBS only.dta')
items.adbs <- colnames(data) #[grep(colnames(data))]
dat <- matrix(data = NA, nrow = dim(data)[1], ncol = dim(data)[2])
for (i in 1:dim(data)[2]) {
  dat[,i] <- c(as.numeric(data[[i]]))
}
colnames(dat) <- items.adbs
```

### 2. only needed for factor correlation difference

model for all items: estimate now so it doesn’t slow down the
algorithm later - it won’t change anyway

```
library(lavaan)
full.model <- 'pros =~ adbs01 + adbs02 + adbs04 + adbs07 + adbs09 + adbs14 +
      adbs16 + adbs17 + adbs19 + adbs20 + adbs22 + adbs23 + adbs26
      cons =~ adbs03 + adbs05 + adbs06 + adbs08 + adbs10 + adbs11 + adbs12 + 
      adbs13 + adbs15 + adbs18 + adbs21 + adbs24 + adbs25'
fit.full <- cfa(model = full.model, 
                data = dat, 
                ordered = items.adbs,
                estimator = 'WLSMV',
                std.lv = TRUE)
out.full <- capture.output(summary(fit.full, fit.measures=TRUE, standardized=TRUE))
std.full <- standardizedSolution(fit.full)
```

## 3. set parameters

these can vary depending on your data

```
nitems <- 10 # number of items in the short scale
iter <- 50  # number of iterations
ants <- 60  # number of ants
evaporation <- 0.8 # evaporation
```

### 4. set summary files

```
summaryfile <- c("E:/ACO/Daten/fitinfo.txt") # lists resutls of all runs
summaryfile2 <- c("E:/ACO/Daten/results.csv") # lists results of best solution
loadings <- c("E:/ACO/Daten/factor_loadings.csv") # can also be added to the summaryfile2; that can be specified in the function
```

### 5. make sure the function refers to the correct data / items

e.g. “item.vector <- items.adbs” “data = dat” FUNCTION START:

```
antcolony <- function(evaporation, items.adbs, nitems, iter, ants, summaryfile, summaryfile2, loadings) {

  best.pheromone <- 0
  best.so.far.pheromone <- 0
  item.vector <- items.adbs
  item.vector1 <- items.adbs[c(1,2,4,7,9,14,16,17,19,20,22,23,26)]
  item.vector2 <- items.adbs[c(3,5,6,8,10,11,12,13,15,18,21,24,25)]
  
  #creates the table of initial pheromone levels.
  include <- rep(2, length(items.adbs))
  #per factor
  include1 <- rep(2, length(item.vector1))
  include2 <- rep(2, length(item.vector2))
  
  #puts initial best solution (all items selected).
  best.so.far.solution <- include
  
  #creates a list to store factors.
  items.adbs1 <- items.adbs[c(1,2,4,7,9,14,16,17,19,20,22,23,26)]
  items.adbs2 <- items.adbs[c(3,5,6,8,10,11,12,13,15,18,21,24,25)]
  
  #starts counting the iterations
  count <- 1
  
  #starts counting continuous runs regardless of result.
  run <- 1
  
  #defines initial solutions.
  previous.solution <- include
  
  #starts loop through iterations.
  while (count <= iter) { 
    
    #sends a number of ants per time.
    ant  <- 1
    while (ant <= ants) {
      
      #################################################################
      ###### ADJUST: This is the lavaan model for the short scale
      ###### adjust as necessary
      adbs <- NULL

      #selects the items for a short form for the factor
      #item selection for 2-factor model:
      positions1 <- is.element(item.vector1, items.adbs1)
      prob1 <- include1[positions1]/ sum(include1[positions1])
      positions2 <- is.element(item.vector2, items.adbs2)
      prob2 <- include2[positions2]/ sum(include2[positions2])
      items1 <- sample(items.adbs1, size = 5, replace = F, prob1)
      items2 <- sample(items.adbs2, size = 5, replace = F, prob2)
      items <- cbind(items1, items2)
      #stores selected items 
      selected.items1 <- items1
      selected.items2 <- items2
      selected.items <- items
            
      # specifies CFA model
      adbs <- paste("pros =~", paste(selected.items1, collapse = " + "), 
                    sep = "\n ", 
                    "cons =~", paste(selected.items2, collapse = " + "))
     
      #creates a 0/1 vector of the same length of the long form indicating
      #whether an item was selected or not for the short form.
      select.indicator <- is.element(item.vector, selected.items)
      notselect.indicator <- (select.indicator == FALSE)
      
      # estimates CFA model
      fit <- cfa(model = adbs, 
                 data = dat, 
                 ordered = items.adbs,
                 estimator = 'WLSMV',
                 std.lv = TRUE)

      # save the complete lavaan ouput
      out <- capture.output(summary(fit, fit.measures=TRUE, standardized=TRUE))
      std <- standardizedSolution(fit)
      
      #################################################################
      ###### ADJUST: choose / adjust optimization criteria for the pheromone
      # reads the fit statistics (CFI, RMSEA)
      CFI <- fitMeasures(fit, "cfi")[[1]]
      RMSEA <- fitMeasures(fit, "rmsea")[[1]]

      # optimization #1: model fit
      phi.CFI <- 1/(1+exp(95-100*CFI))
      phi.RMSEA <- 1-(1/(1+exp(5-100*RMSEA)))
      phi.fit <- (phi.CFI + phi.RMSEA)/2
      
      # optimization #2: omega
      sum.lam <- sum(std[1:nitems, 4])^2
      sum.eps <- sum(1-std[1:nitems, 4]^2)
      omega <- sum.lam/(sum.lam + sum.eps)
      phi.lam <- 1/(1+exp(9-10*omega))
      
      # extract factor loadings
      min.load <- min(std[1:nitems, 4])
      max.load <- max(std[1:nitems, 4])
      mean.load <- mean(std[1:nitems, 4])
      all.load <- paste(std[1:nitems, 4], collapse = "/")
      
      # optimization #3: correlation short scale and original scale
      items.long <- rowSums(dat[, items.adbs])
      items.short <- rowSums(dat[, selected.items])
      scales.sums <- cbind(items.long, items.short)
      cor.sc <- cor(scales.sums, use="p")
      cor.scales <- cor.sc[1,2]
      phi.cor <- 1/(1+exp(85-100*cor.scales))
      
      #extract factor correlations:
      psi.full <- lavInspect(fit.full, what = "std")$psi
      psi <- lavInspect(fit, what = "std")$psi
      fc.full <- psi.full[2, 1]
      fc <- psi[2, 1]
      
      # optimization #4: factor correlation difference
      cor.diff <- abs(fc.full - fc)
      phi.fc <- 1-(1/(1+exp(3-100*cor.diff)))
      
      # max f(x), optimize = maximize
      pheromone <- phi.fit + phi.lam + phi.cor + phi.fc
      
      #################################################################
      ###### ADJUST: make sure this contains everything you want to see in your summary file
      #saves information about the selected items and the model fit they generated.
      fit.info <- matrix(c(select.indicator, run, count, ant, 
                           CFI, RMSEA, phi.CFI, phi.RMSEA, phi.fit,
                           omega, phi.lam,
                           cor.scales, phi.cor,
                           fc, cor.diff, phi.fc,
                           pheromone, round(include1,2), round(include2,2)), 1)
      
      write.table(fit.info, file = summaryfile, append = T,
                  quote = F, sep = ";", row.names = F, col.names = F)
      
      #adjusts count based on outcomes and selects best solution.
      if (pheromone >= best.pheromone) {
        
        #################################################################
        ###### ADJUST: if you use different optimization criteria
        
        # updates solution.
        best.solution <- select.indicator
        best.pheromone <- pheromone
        
        # updates best model fit
        best.RMSEA <- RMSEA
        best.CFI <- CFI
        best.phi.CFI <- phi.CFI
        best.phi.RMSEA <- phi.RMSEA
        best.phi.fit <- phi.fit
        
        # updates best factor loadings
        best.min.load <- min.load
        best.mean.load <- mean.load
        best.max.load <- max.load
        best.all.load <- all.load
        
        # update best omega
        best.omega <- omega
        best.phi.lam <- phi.lam
        
        # update best scale correlation
        best.cor.scales <- cor.scales
        best.phi.cor <- phi.cor
        
        # update best factor correlation
        best.fc <- fc
        best.cor.diff <- cor.diff
        best.phi.fc <- phi.fc
        
      } 
      
      #Move to next ant.
      ant <- ant + 1
      
      #ends loop through ants.
    }
    
    #adjusts pheromone only if the current pheromone is better than the previous.
    if (best.pheromone > best.so.far.pheromone) {
      
      #implements pheromone evaporation.
      include <- c(include1, include2) * evaporation
      
      #adjusts the pheromone levels.
      include.pheromone <- best.solution * best.pheromone * run * 0.2
      
      #updates pheromone.
      include <- include + include.pheromone
      #################################################################
      ###### ADJUST: for item selection per factor - not necessary for 1-factor model
      include1 <- include[c(1,2,4,7,9,14,16,17,19,20,22,23,26)]
      include2 <- include[c(3,5,6,8,10,11,12,13,15,18,21,24,25)]
      
      
      #################################################################
      ###### ADJUST: if you use different optimization criteria
      
      # updates best so far solution and pheromone.
      best.so.far.solution <- best.solution
      best.so.far.pheromone <- best.pheromone
      best.so.far.RMSEA <- best.RMSEA
      best.so.far.CFI <- best.CFI
      best.so.far.phi.CFI <- best.phi.CFI
      best.so.far.phi.RMSEA <- best.phi.RMSEA
      best.so.far.phi.fit <- best.phi.fit
      
      best.so.far.min.load <- best.min.load
      best.so.far.mean.load <- best.mean.load
      best.so.far.max.load <- best.max.load
      best.so.far.all.load <- best.all.load
      
      best.so.far.omega <- best.omega
      best.so.far.phi.lam <- best.phi.lam
      
      best.so.far.cor.scales <- best.cor.scales
      best.so.far.phi.cor <- best.phi.cor
      
      best.so.far.fc <- best.fc
      best.so.far.cor.diff <- best.cor.diff
      best.so.far.phi.fc <- best.phi.fc
      
      #re-starts count.
      count <- 1
      
      #end if clause for pheromone adjustment.
    } else {
      
      #advances count.
      count <- count + 1
    }
    
    #ends loop.
    run <- run + 1
  }
  
  #################################################################
  ###### ADJUST: make sure this contains everything you want to see in your summary file
 title.final.solution = matrix(c("CFI", "RMSEA", "phi.CFI", "phi.RMSEA", "phi.fit",
                                 "omega", "phi.lam",
                                 "cor.scales", "phi.cor",
                                 "factor.cor", "cor.diff", "phi.fc",
                                 "pheromone", item.vector), 1)
    
 write.table(title.final.solution, file = summaryfile2, append = T,
                quote = F, sep = ";", row.names = F, col.names = F)
 
 #save factor loadings
 title.factor.load <- matrix(c("min.load", "mean.load", "max.load", "all.load", item.vector), 1)
 write.table(title.factor.load, file = loadings, append = T, quote = F,
             sep = ";", row.names = F, col.names = F)
 
 # Compile a matrix with the final solution.
 final.solution <- matrix(c(best.so.far.CFI, best.so.far.RMSEA, best.so.far.phi.CFI, best.so.far.phi.RMSEA, best.so.far.phi.fit,
                            best.so.far.omega, best.so.far.phi.lam,
                            best.so.far.cor.scales, best.so.far.phi.cor,
                            best.so.far.fc, best.so.far.cor.diff, best.so.far.phi.fc,
                            best.so.far.pheromone, best.so.far.solution), 1)
  
 write.table(final.solution, file = summaryfile2, append = T,
             quote = F, sep = ";", dec = ",", row.names = F, col.names = F)
 
 factor.load <- matrix(c(best.so.far.min.load, best.so.far.mean.load, best.so.far.max.load, best.so.far.all.load, best.so.far.solution), 1)
 write.table(factor.load, file = loadings, append = T, quote = F, 
             sep = ";", dec = ",", row.names = F, col.names = F)
 
  return(best.so.far.solution)
}
```

### 6. make sure your parameters and files are correctly specified

run the function

```
library(lavaan)
library(psych)

short <- antcolony(evaporation, items.adbs, nitems, iter, ants, summaryfile, summaryfile2, loadings)
```
