# Supplementary material for: Application of the ant colony optimization algorithm for the construction of a short version of the German alcohol decisional balance scale
Source: Sci Rep. 2025 Jul 25;15:27122. doi: 10.1038/s41598-025-12087-3 (PMC12297269; doi:10.1038/s41598-025-12087-3)
Supplement: Supplementary file 2 — Supplementary Material 2 [file 41598_2025_12087_MOESM2_ESM.docx]

**Supplementary Table S1**

*Measurement invariance testing for the full ADBS scale across studies and gender*

| Model | | χ^2^(df) | CFI | RMSEA | χ^2^(Δdf) | ΔCFI |
| --- | --- | --- | --- | --- | --- | --- |
| Studies | |  |  |  |  |  |
|  | 1 configural | 2936.8 (894) | .951 | .069 |  |  |
|  | 2 metric | 3167.6 (1046) | .949 | .065 | 364.9 (152) | .002 |
|  | 3 scalar | 3625.5 (1094) | .939 | .069 | 480.6 (48) | .010 |
| Gender | |  |  |  |  |  |
|  | 1 configural | 1487.9 (70) | .955 | .065 |  |  |
|  | 2 metric | 1195.1 (99) | .965 | .048 | 196.1 (29) | .010 |
|  | 3 scalar | 1106.9 (107) | .968 | .044 | 102.9 (8) | .003 |

Note. CFI = Comparative Fit Index; RMSEA = Root Mean Square Error of Approximation.

**Supplementary Table S2**

*Measurement invariance testing for the ACO-P scale across studies and gender*

| Model | | χ^2^(df) | CFI | RMSEA | χ^2^(Δdf) | ΔCFI |
| --- | --- | --- | --- | --- | --- | --- |
| Studies | |  |  |  |  |  |
|  | 1 configural | 213.1 (102) | .995 | .047 |  |  |
|  | 2 metric | 332.7 (158) | .992 | .047 | 128.5 (56) | .003 |
|  | 3 scalar | 647.3 (174) | .977 | .074 | 219.6 (16) | .015 |
| Gender | |  |  |  |  |  |
|  | 1 configural | 203.9 (68) | .990 | .062 |  |  |
|  | 2 metric | 222.7 (96) | .989 | .055 | 39.9 (28) | .001 |
|  | 3 scalar | 240.2 (104) | .990 | .050 | 12.8 (8) | .001 |

Note. CFI = Comparative Fit Index; RMSEA = Root Mean Square Error of Approximation.

**Supplementary Table S3**

*Measurement invariance testing for the ACO-C scale across studies and gender*

| Model | | χ^2^(df) | CFI | RMSEA | χ^2^(Δdf) | ΔCFI |
| --- | --- | --- | --- | --- | --- | --- |
| Studies | |  |  |  |  |  |
|  | 1 configural | 269.1 (129) | .994 | .047 |  |  |
|  | 2 metric | 383.7 (191) | .991 | .045 | 125.1 (62) | .003 |
|  | 3 scalar | 573.4 (209) | .984 | .059 | 144.5 (18) | .007 |
| Gender | |  |  |  |  |  |
|  | 1 configural | 211.7 (86) | .995 | .044 |  |  |
|  | 2 metric | 245.7 (117) | .995 | .038 | 29.4 (31) | < .001 |
|  | 3 scalar | 248.3 (126) | .995 | .036 | 14.8 (9) | < .001 |

Note. CFI = Comparative Fit Index; RMSEA = Root Mean Square Error of Approximation.
